# Influence Mechanism of Water Content and Compaction Degree on Shear Strength of Red Clay with High Liquid Limit

**DOI:** 10.3390/ma17010162

**Published:** 2023-12-28

**Authors:** Xuemao Feng, Jidong Teng, Hongwei Wang

**Affiliations:** 1 School of Civil Engineering, Central South University, Changsha 410083, China; xfzfxm1984@163.com; 2 Guangxi Xinfazhan Communication Group Co., Ltd., Guangxi Zhuang Autonomous Region, Nanning 530029, China; gxu_whw@163.com

**Keywords:** high liquid limit, red clay, compaction degree, water content, shear strength, influence mechanism

## Abstract

To investigate the influencing factors and mechanisms of shear strength of red clay with a high liquid limit, which was selected at different milepost locations based on the Nanning Bobai Nabu Section Project of the Nanning Zhanjiang Expressway, the basic physical properties of red clay were determined using a liquid plastic limit test, compaction test, inductively coupled plasma optical emission spectrometer (ICP-OES), and X-ray fully automatic diffractometer (XRD). Red clay with a high liquid limit was selected. Furthermore, the direct shear test was used to study the effect of different water contents and compaction degrees on the shear strength. The experimental results demonstrate that under the same compaction degree, the shear stress of the soil sample increases significantly with an increase in normal stress, and the greater the water content, the smaller the shear stress of the soil sample. At 200 kPa, the shear strength of soil samples with 24% water content is 57%, 46%, and 35% of the shear strength of soil samples with 15% water content under different compaction degrees(K) of 86%, 90%, and 93%, respectively. Under the same moisture content, the shear stress of the soil sample shows an increasing trend with an increase in the degree of compaction, and the greater the compaction degrees, the greater the shear stress of the soil sample. The cohesion *c* and internal friction angle *φ* of soil samples increase with an increase in the compaction degree, but the increase in cohesion *c* is also affected by the water content. Under the condition of low water content, the cohesion *c* of soil samples can be increased by 1.06 times when the water content is 15% and by 0.47 times when the water content is 18%. Under the condition of high water content, the cohesion *c* of soil samples with 21% water content only increases by 0.3 times, and that with 24% water content only increases by 0.35 times.

## 1. Introduction

Red clay is widely distributed in southern China, mainly in areas such as Guangxi, Guizhou, Yunnan, Guangdong, and Hunan. Its high natural moisture content, high content of fine particles, and strong water sensitivity lead to different characteristics between mechanical parameters and physical indicators compared to silt and sand [1,2,3]. Due to the hilly and mountainous terrain in the southern region of China, highways face a large number of red clay excavation and filling problems during the construction process. However, in humid and rainy climates, it is difficult to reduce the natural moisture content of red clay to near the optimal moisture content through sun exposure to achieve the corresponding compaction control standards. However, using improvement or replacement methods can cause significant cost increases and environmental pollution [4,5,6]. Therefore, roadbed construction in red clay areas can easily lead to roadbed settlement, shoulder collapse, slope instability, and other engineering defects [7,8,9]. Therefore, how to efficiently handle and apply this type of soil is an urgent engineering problem that needs to be solved.

The U.K. stipulates that the moisture content of roadbed filling should be lower than the plastic limit of the soil, while the U.S. allows the moisture content of roadbed filling to be 1.2 times the plastic limit under the condition that the liquid limit of clay filling does not exceed 50%. Japan has different compaction control standards based on different soil properties, while China stipulates that while ensuring the strength and deformation of the roadbed, for special soil embankments in special climate areas, the compaction standards and filling water content requirements can be appropriately reduced. At the same time, soil with a liquid limit greater than 50% and a plasticity index greater than 26 cannot be directly used as roadbed filler [10,11]. In summary, there is no unified standard for compaction control of fill embankments, both domestically and internationally. There are no specific reference opinions on the relaxation requirements for compaction and moisture content, and the engineering characteristics of red clay fillers vary in different regions [12,13]. Therefore, it is urgent to start from the basic needs of filling red clay embankments, exploring the relationship between the basic physical and mechanical indicators of Guangxi red clay and the changes in compaction and moisture content and providing guidance for engineering construction [14,15,16].

At present, domestic and foreign experts and scholars have conducted extensive research on the mechanical properties of red clay and achieved certain results. Zhao et al. added waste tires to red clay and conducted triaxial tests to study the changes in the strength parameters of improved red clay at different dosages. At the same time, scanning electron microscopy technology was used to analyze the microstructure characteristics of red clay, and mercury intrusion experiments were used to obtain the pore structure characteristics of improved red clay at different dosages. The effects of factors such as the microstructure and static indicators of the filler were comprehensively considered [17]. Chen et al. used triaxial tests to study the effects of dry density and moisture content of saturated and unsaturated remolded red clay on soil resistance, the influence of shear strength. The experimental results show that the quadratic polynomial fitting curve between the cohesion and dry density of saturated remolded red clay under the same optimal moisture content is concave, while the curve between the cohesion and dry density of unsaturated soil is convex [18]. Chen et al. analyzed the change rule of cracks and shear strength under cyclic wet–dry conditions and established the functional relationship between crack rate and shear strength index of red clay [19,20]. Qi et al. researched the mechanical properties of red clay after indoor wet–dry cycles through triaxial consolidation drainage and direct shear tests [21]. Through the unconfined compressive strength (UCS), wetting–drying (WD), and low-temperature nitrogen adsorption tests, Wang et al. found that the failure mechanism in the UCS test varies depending on the dry density [22]. Chen conducted shear tests on soil samples from different weather conditions and depths by embedding sensors inside the red clay slope and analyzing the characteristics and evolution process of slope failure [23]. A Pham micromechanical model for predicting the shear strength of red clay was constructed based on stress equilibrium conditions and multiphase interaction relationships, and its practicality and effectiveness were demonstrated using experimental data [24]. Although many scholars and experts have conducted extensive research on red clay, the characteristics of high-liquid-limit red clay vary in different regions. How to quickly obtain the shear strength parameters of red clay in engineering and reveal its influencing factors and mechanisms from a microscopic perspective is an urgent research direction that needs to be explored.

This study relies on the Nanning to Bobai Nabu section project of the Nanning Zhanjiang Expressway in Guangxi, focusing on the engineering problems encountered in the construction of red clay areas: what are the relaxed requirements for compaction and moisture content, and how much are they relaxed? Selecting red clay at different mileage stake locations, conducting a series of indoor experiments to explore the physical properties of the red clay foundation, deeply understanding the three-phase interaction law, and conducting direct shear tests on unsaturated red clay to study the impact of different water contents and compaction on the shear strength of high-liquid-limit red clay. Based on this, the impact of water content and compaction on the construction of red clay roadbeds is judged and explored, providing suggestions for roadbed construction in red clay areas, and applying it to the construction of highways in Guangxi.

## 2. Physical Properties of Red Clay

To facilitate sample comparison, samples at K18 + 480, K21 + 100, K21 + 700, and K22 + 980 (commonly referred to as a mileage sign, where K represents the entire kilometer stake number and the number on it represents the distance of how many kilometers from the starting point of the line) stake numbers were selected for test research after field geological survey.

### 2.1. Liquid Plastic Limit Test

**Figure 1:**
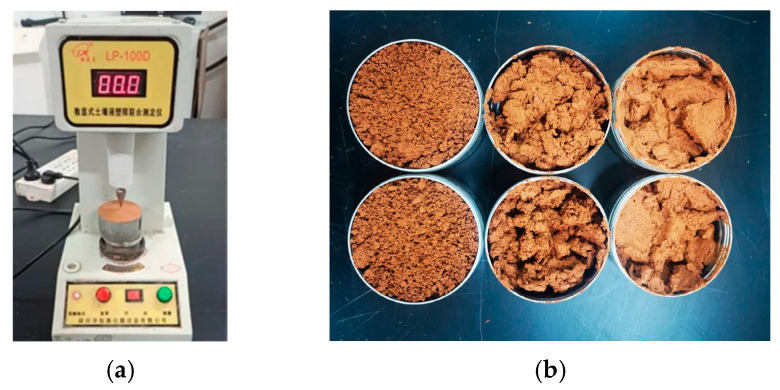
Liquid plastic limit test and sample. (**a**) Liquid-plastic combine tester, Digital display soil liquid plastic limit joint measuring instrument, which is made by Shaoxing Expanding Instrument and Equipment Co., Ltd., Shangyu, China; (**b**) Soil sample.

Liquid and plastic limits are important physical property indices of cohesive soil, which reflect the influence of the presence of water in soil samples on the properties of soil samples. Therefore, the accurate determination of liquid and plastic limit indices of soil has a great deal of significance for engineering. The liquid plastic limit joint measurement method refers to the method of measuring the sinking depth of a cone under three different water content states of a soil sample, drawing a curve of the relationship between the sinking depth of the cone and the water content, and then obtaining the liquid and plastic limit values from the curve. In the experiment, a liquid plastic limit joint tester was used to measure the liquid and plastic limits of four pile number soil samples, and based on this, the soil samples were classified to determine whether they were high-liquid-limit clay. The liquid–plastic combine tester and soil sample are shown in Figure 1. Specific results are shown in Table 1. According to highway geotechnical testing regulations [25], if the liquid limit is greater than 50% and the plasticity index is greater than 0.73 wL-20, where wL represents liquid limit, it is determined to be a high-liquid-limit clay. Therefore, samples at K21 + 100 were selected for subsequent research.

### 2.2. Composition Analysis of Samples

**Figure 2:**
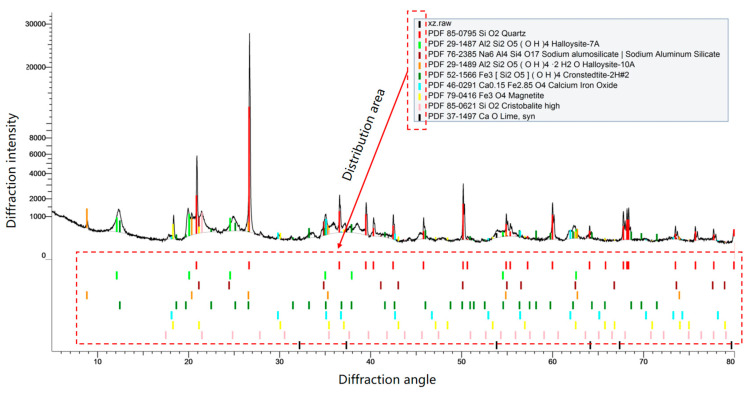
Test results of X-ray fully automatic diffractometer.

The strength of red clay is primarily due to the mutual attraction between soil particles and the cementation of iron oxide. The action mechanism of ferric oxide cement has not been thoroughly studied due to its high clay content and the complex structure between particles. Therefore, the ICP-OES and XRD can be used to observe the influence of soil particle iron content on microcontact, macro-physical, and mechanical properties [26]. The ICP-OES can be used to perform qualitative analysis according to the spectral line eigenvalue formed by the atomic energy level structure of the required test element and quantitative analysis according to the emission intensity obtained by the atomic concentration of the required test element [27,28]. The XRD is widely used in materials science and other fields and can be used to obtain sample composition and content after qualitative and quantitative analysis of materials [29,30]. The test results of the ICP-OES and the XRD spectrum curves are shown in Table 2 and Figure 2 respectively. According to the test results in Table 2, the content of Al in red clay is the highest, followed by the content of Fe, followed by the content of K, and the content of other elements is relatively small. It can be seen from Table 2 and Figure 2 that the main forms are quartz (SiO_2_), iron oxide (Fe_3_O_4_), Fe_3_[Si_2_O_5_](OH)_4_, kaolinite (Al_2_ [Si_2_O_5_](OH)_4_), Al_2_ [Si_2_O_5_](OH)_4_2H_2_O, etc. As the quartz particles are large and non-plastic, and the unit layers of the kaolinite structure contain strong bonding force, the unit layers of the structure will not disperse, and the lattice activity is small. Therefore, the mineral particles can form a stable aggregate structure with a stable crystalline framework, which makes the red clay particles closely connected internally, showing the characteristics of low compressibility and high strength.

### 2.3. Compaction Test

**Figure 3:**
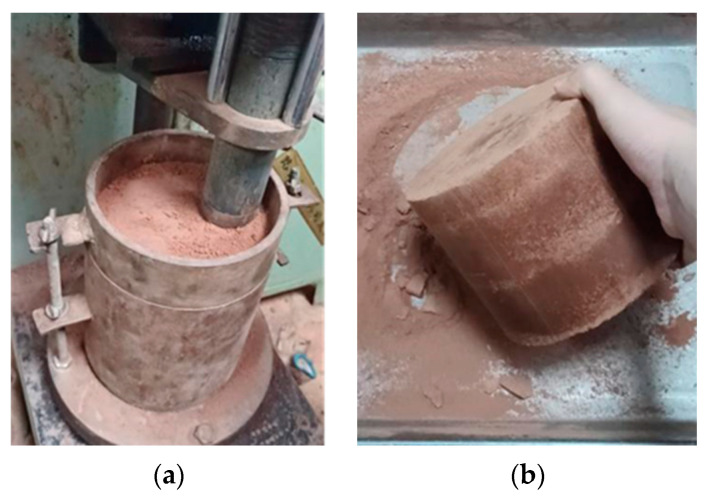
Compaction test and sample. (**a**) Compaction test; (**b**) Soil sample.

**Figure 4:**
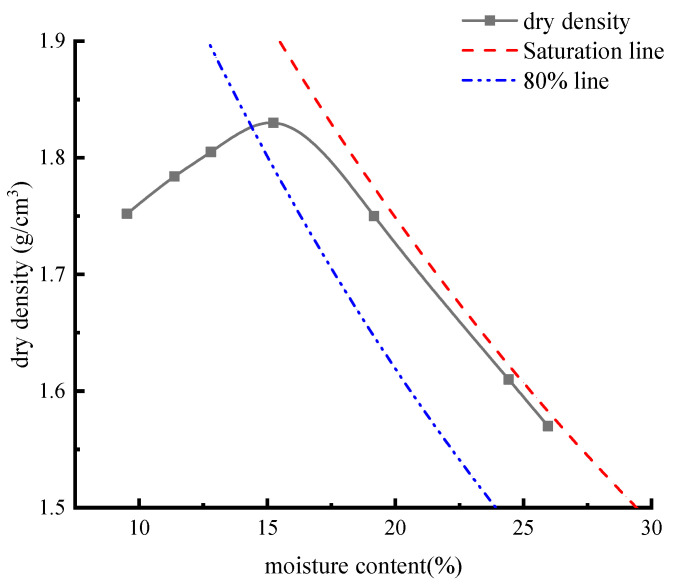
Compaction test result curve.

The maximum dry density and optimal moisture content of soil samples are important indices to determine the degree of subgrade compaction; therefore, it is important to accurately obtain the data to ensure the safety of the project. According to highway geotechnical testing regulations, we use heavy-duty compaction, with a hammer bottom diameter of 5 cm, a hammer mass of 4.5 kg, a drop distance of 45 cm, and a unit volume compaction work of 2682.7 kJ/m^3^. The compaction is divided into three layers, with 98 blows per layer. Because the soil sample at K21 + 100 is a high-liquid-limit red clay, a wet weight compaction test should be used. The compaction test and sample are shown in Figure 3. The specific test results were shown in Table 3. The compaction curve is drawn according to the data in the Table 3, as shown in Figure 4. According to the data corresponding to the peak point in Figure 4, the maximum dry density is 1.83 g/cm^3^, and the optimal moisture content is 15.3%.

## 3. Test Protocol

### 3.1. Test Instrument

**Figure 5:**
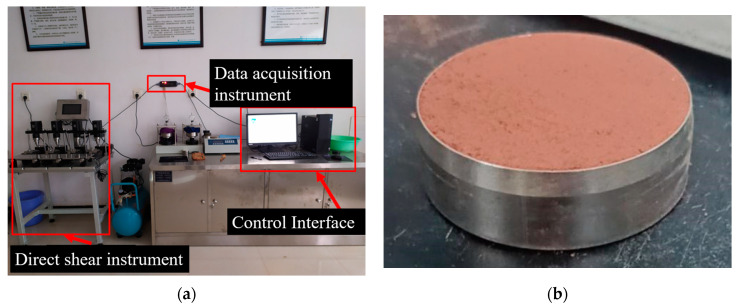
Fully automatic quadruple shear tester and ring knife sample. (**a**) Fully automatic quadruple shear tester; (**b**) Soil sample.

The experimental instrument adopts an AZJ-4 fully automatic four-link direct shear instrument produced by Nanjing Zhilong Technology Co., Ltd., Nanjing, China. This system is equipped with electric pneumatic loading, with a control range of shear rate of 0–5 mm/min, vertical load of 0–400 kPa, and shear load range of 0–1.2 kN. The test adopts fully automatic computer control collection and processing and can perform various shear tests such as slow shear, fast shear, fixed fast shear, and reciprocating shear. The purpose of applying vertical pressure is to measure the shear strength indicators of soil samples under different positive pressures—internal friction angle and cohesion, providing necessary information for estimating the bearing capacity of the foundation, evaluating the stability of the foundation and soil slope, and calculating the soil pressure of retaining walls. The inner diameter of the ring knife used is 61.8 mm, the height is 20 mm, and the volume is 59.9925 cm^3^ [31]. The test instrument and ring cutter soil sample are shown in Figure 5.

### 3.2. Sample Preparation

The shear strength will be affected by water content, compaction degrees, and normal stress; therefore, the control variable method can be used to study the effect of different compactness or moisture content on shear strength [32]. According to the optimal moisture content of soil samples, four moisture contents of 15%, 18%, 21%, and 24% were selected, and three compaction degrees of 86%, 90%, and 93% were selected according to the compaction degree requirements of subgrade filling. In general, the natural moisture content of red clay is close to 30%; however, through compaction tests, we can obtain an optimal moisture content of 15%. In actual construction, it is difficult to quickly reduce the soil moisture content to near the optimal moisture content, which affects the construction period. Therefore, we investigated whether red clay can be compacted on the wet side with the optimal moisture content. Therefore, we set four variables: 15%, 18%, 21%, and 24% moisture content to study the changes in the shear strength of red clay under the four different moisture content states. In addition, it is difficult to achieve the specified compaction degree by rolling red clay on the wet side with the optimal moisture content. The compaction standard for highway embankments is 93%; therefore, three variables of 93%, 90%, and 86% moisture content were set to study the changes in shear strength of red clay under three different compaction degrees. The main purpose of this study is to ensure that the shear strength of red clay meets the standard and determine whether the construction moisture content and compaction requirements of red clay roadbed can be appropriately relaxed. Specific grouping were shown in Table 4. We used distilled water to prepare soil samples with different water contents, used fresh bags to seal them in separate bags, and placed them in a moisturizing tank for 24 h. A total of 4×4×3=48 samples need to be prepared for the entire series of direct shear tests.

According to Equation (1), the relationship between the target dry density, compactness, and compaction test data can be obtained using the following equation:

(1) $$\rho_{d}=K\times \rho_{d\max}$$

where *ρ_d_* is actual dry density(g/cm^3^) and *K* is compaction degrees of 86%, 90%, 93%. *ρ_dmax_* is maximum dry density(g/cm^3^) obtained using the compaction test.

Using Equation (2), we can calculate the wet density of soil samples at each specific compaction degree and moisture content:

(2) $$\rho_{w}=\rho_{d}\times \left(1+\omega \right)$$

where *ρ_w_* is the wet density(g/cm^3^), and *w* is moisture content.

From Section 3.1, it can be seen that the volume of the ring cutter is 60 cm^3^; therefore, we combined Equations (1) and (2) to obtain the required soil mass for each working condition, accurately weighed and compacted into shape. The final wet soil mass *m* is shown in Equation (3) [33,34].

(3) $$m=K\times \rho_{d\max}\times (1+w)\times 60$$

### 3.3. Test Procedure

The specific steps of the direct shear test are as follows: ① First, Vaseline is applied to the inner wall of the ring cutter to ensure smooth demolding; ② red clay samples with different compaction degrees and moisture contents are prepared using the compaction method; ③ the ring knife sample is placed in the shear box with a piece of permeable stone and wax paper placed on both sides to prevent moisture loss, and a fixed pin is inserted to temporarily prevent the relative displacement of the upper and lower boxes; ④ the edge of the ring knife is moved upward, the top edge of the ring knife is aligned with the shallow groove on the top surface of the upper box, and a bulldozer plug is used to gently press it into the shear box, cover the box cover, and adjust the vertical and horizontal pressure gauges; ⑤ the shear rate is adjusted to 0.800 mm/min, four sets of vertical pressures are set in the control system to 50 kPa, 100 kPa, 150 kPa, and 200 kPa, stopping when the shear deformation is set to 8 mm; and ⑥ data are recorded.

## 4. Analysis of Test Results

### 4.1. Stress–Strain Deformation

**Figure 6:**
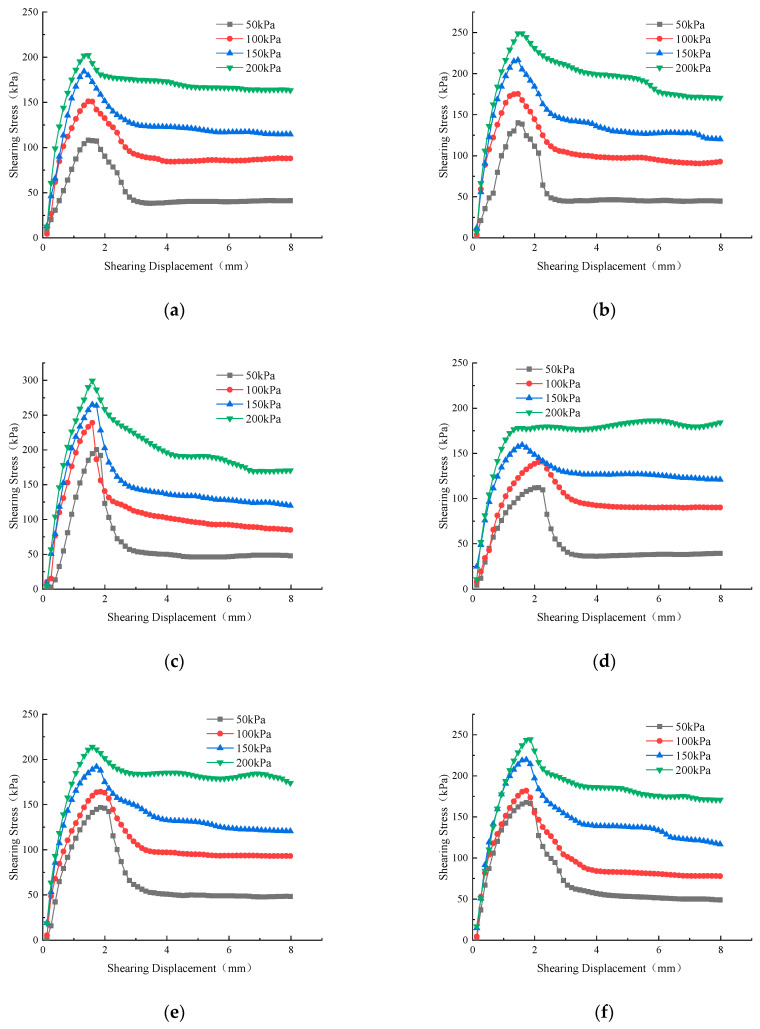

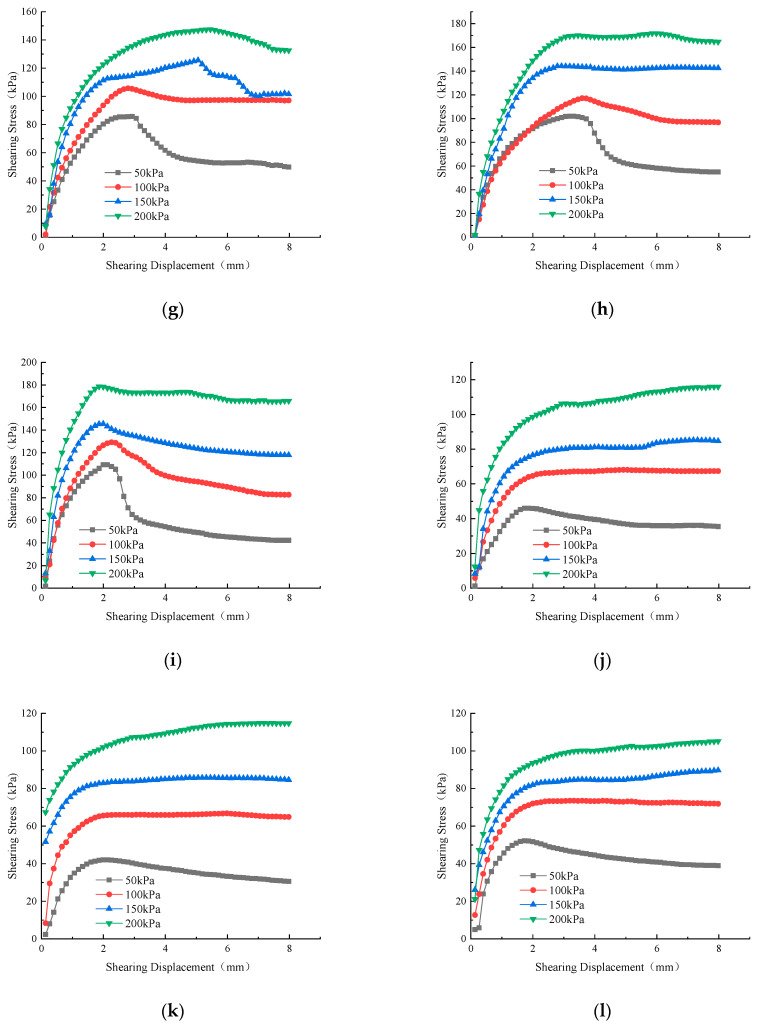
Stress–strain deformation. (**a**) *w* = 15%, *K* = 86; (**b**) *w* = 15%, *K* = 90; (**c**) *w* = 15%, *K* = 93; (**d**) *w* = 18%, *K* = 86; (**e**) *w* = 18%, *K* = 90; (**f**) *w* = 18%, *K* = 93; (**g**) *w* = 21%, *K* = 86; (**h**) *w* = 21%, *K* = 90; (**i**) *w* = 21%, *K* = 93; (**j**) *w* = 24%, *K* = 86; (**k**) *w* = 21%, *K* = 93; (**l**) *w* = 24%, *K* = 86.

Figure 6 shows the shear strength vs. displacement curves of the compacted red clay samples under different vertical pressure conditions and different working conditions. By analyzing the variation patterns of red clay samples with w = 15% and K = 0.86 under different vertical pressures, it was found that the peak shear strength of the red clay samples also increases with vertical pressure. The peak shear strength was 115 kPa when the load was 50 kPa, 155 kPa when the load was 100 kPa, 180 kPa when the load was 150 kPa, and 210 kPa when the load was 200 kPa. As the moisture content increased, the peak shear strength of the red clay sample continued to decrease. When the moisture content was 15%, the peak shear strength was 200 kPa. The shear strength was 170 kPa at a moisture content of 18%, 115 kPa at a moisture content of 21%, and 53 kPa at a moisture content of 24%. When the moisture content was between 15% and 24%, as the moisture content increased, its shear resistance also decreased, and the displacement curve of the failure point moved to the right, transforming the failure mode from stress softening to stress hardening. As the compaction degree increased, the shear peak strength of the red clay sample was enhanced. When the compaction degree was 0.86, the peak shear strength was 110 kPa. When the compaction degree was 0.90, the shear strength was 130 kPa, and when the compaction degree was 0.93, the shear strength was 210 kPa. When the compaction degree was between 0.86 and 0.93, as the compaction degree increased, its shear resistance also increased, and the displacement curve of the failure point moved to the right [35].

### 4.2. Effect of Water Content on Shear Strength of Red Clay

**Figure 7:**
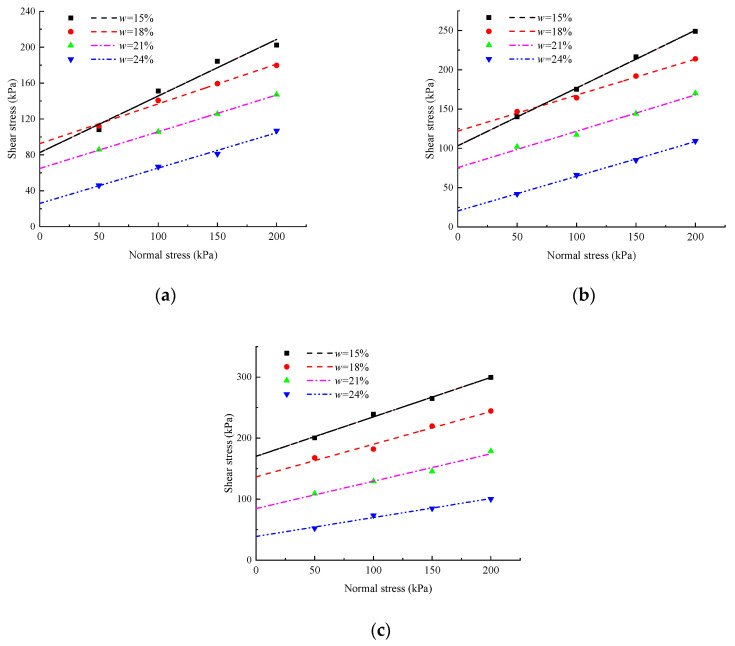
Shear strength of specimens with different moisture content. (**a**) K = 86%; (**b**) K = 90%; (**c**) K = 93%.

The shear strength of soil reflects its mechanical properties, while the strength mainly depends on the connection between particles and the cementation effect of free iron oxide. Under the same compaction degrees, the relationship between shear stress and normal stress of soil samples with different water contents is shown in Figure 7. It can be seen that under the same compaction degrees, the shear stress of the soil sample shows an obvious growth trend with an increase in normal stress, and the greater the water content, the smaller the shear stress of the soil sample. Under the condition of 200 kPa, the shear strength of samples with 24% water content under different compaction degrees is 57%, 46%, and 35% of the shear strength of samples with 15% water content. When the normal stress is 50 or 100 kPa and the compaction degree is not more than 90%, the difference in shear strength between 15% and 18% water content is very small. However, with an increase in normal stress or compaction degrees, the shear strength of 15% water content will be higher than that of 18% water content. The influence of water content on the shear strength of the soil mass increases with an increase in the compaction degree. The greater the normal stress, the greater the water content, and the greater the weakening effect of the soil shear strength. This shows that under the condition of a certain compaction degree, the change in the shear strength of the sample conforms to the expression of shear strength of cohesive soil proposed by Coulomb. At this time, the shear strength of the soil sample is mainly affected by the normal stress on the direct shear failure surface. Because the *φ* of soil samples is mainly affected by the occlusion between soil particles and the *c* is affected by the cementation between soil particles and the combined water effect, the interlocking effect is mainly related to the particle structure, size, and density of the soil, while the moisture content does not cause significant changes in the microstructure of compacted red clay.

### 4.3. Effect of Compaction Degrees on Shear Strength of Red Clay

**Figure 8:**
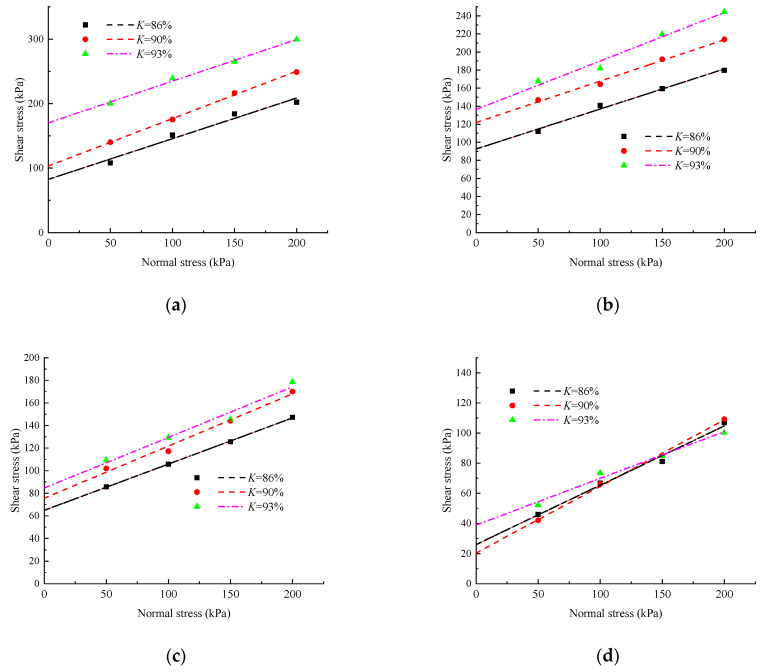
Shear strength of specimens under different compaction degree. (**a**) *w* = 15%; (**b**) *w* = 18%; (**c**) *w* = 21%; (**d**) *w* = 24%.

Under the same water content, the relationship between the shear stress and normal stress of soil samples with different compaction degrees is shown in Figure 8. In Figure 8, under the same moisture content, the shear stress of the samples shows an increasing trend with an increase in compaction degree, and the greater the compaction degree, the greater the shear stress of the soil samples. For samples with water contents of 15% and 18%, changes in compaction degree have a significant impact on the change in shear strength, but for samples with water contents of 21% and 24%, the change in compaction degree has a small impact on the change in shear strength. Fe_3_O_4_ and Fe_3_[Si_2_O_5_](OH)_4_ are more abundant in the sample. Under the optimal water content, the cementation of iron oxide is the strongest, and the *c* between soil particles is the highest, so the sample with 15% water content can bear a greater vertical load.

### 4.4. Shear Strength Index of Red Clay under Different Working Conditions

**Figure 9:**
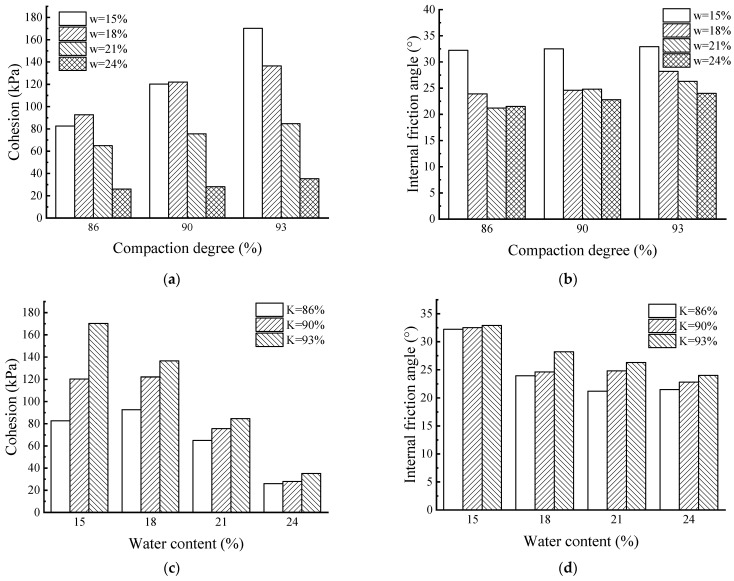
Relationship between cohesion *c*, internal friction angle *φ*, compaction degree, and water content. (**a**) Relationship between cohesion *c* and compaction degree; (**b**) Relationship between internal friction angle φ and compaction degree; (**c**) Relationship between cohesion *c* and water content; (**d**) Relationship between internal friction angle φ and water content.

The shear strength index refers to the internal friction angle *φ* and cohesion *c*. According to the direct shear test results, the statistics of the soil mass shear strength indices under different combinations are shown in Table 5 and Figure 9. In Figure 9a, the *c* of the soil sample increases with an increase in compaction degree, but the increase is affected by the water content. When the soil sample is near the optimal water content, the *c* increases greatly. In Figure 9b, the *φ* of soil sample increases to a certain extent with an increase in compaction degree, indicating that the *φ* is less sensitive to changes in compaction degree.

Under the condition of low water content, the *c* of samples with 15% water content can be increased by 1.06 times, and the *c* of samples with 18% water content can be increased by 0.47 times. Under the condition of high water content, the *c* of samples with 21% water content only increases by 0.3 times, and that with 24% water content only increases by 0.35 times. Cohesive force is affected by water content and compaction degree. With an increase in compaction degree, the soil particles contact more closely, the cementation force and water film bonding force are improved, the friction effect is gradually enhanced, and the *φ* shows a certain increasing trend. When the soil is saturated, the free water content between soil particles increases significantly, and a layer of water film forms around the soil particles to lubricate the dislocation movement between soil particles, greatly reducing the cementation and cohesion *c* between soil particles, leading to a decrease in soil cohesion.

In Figure 9c, under the same compaction degree, the cohesion *c* of soil samples is the largest when they are near the optimal moisture content. When the water content of the sample is higher than the optimal water content, the *c* decreases gradually with an increase in water content. At the same moisture content, the *c* of the samples will increase with an increase in compaction. With an increase in water content, Figure 9d shows that the range of the soil sample decreases. When there is a small amount of water in the soil, particles can directly contact each other and closely occlude. The friction is dominant, and the bonding effect is strong, which is prone to macro shear failure and shows low shear strength. With an increase in water content, on the one hand, because the special double electric layer structure of red clay has strong water absorption, clay minerals in the soil absorb bound water, the bound water film is thin, the particle spacing is small, and the water film bonding force and electrostatic attraction between particles are large. Therefore, the macroscopic performance is that the *c* and shear strength increase accordingly. On the other hand, the cementation of free iron oxide in red clay has a significant impact on its physical and mechanical properties, and the engineering properties have also changed with the change in free iron oxide morphology.

## 5. Discussion

(1) Moisture content, compaction degree, and vertical pressure have important effects on the stress–strain relationship of red clay. Under certain conditions of moisture content and compaction degree, when the vertical pressure exceeds 100 kPa, the influence of vertical pressure on the initial slope of the stress–strain of red clay gradually decreases. In other words, as the vertical pressure increases, the influence of vertical pressure on the elastic modulus of red clay gradually decreases. This requires us to control the minimum vertical pressure of red clay in the research process related to red clay. Under certain conditions of moisture content and vertical pressure, a high degree of compaction can increase the peak value of the stress–strain curve of red clay, but it has little effect on the numerical value of the horizontal section at the end of the stress–strain curve. Under certain conditions of compaction and vertical pressure, the trend of moisture content change is negatively correlated with the peak changes in red clay and the stress–strain curve. Moreover, as the moisture content increases, the peak value of red clay becomes less obvious, and the trend of change in the latter half of the stress–strain curve becomes more gentle. When conducting research on red clay, special attention should be paid to the moisture content, compaction degree, and vertical pressure of the test components.
(2) The dry season is a critical period for highway construction, and the duration of the dry season varies in different regions. Guangxi has a tropical and subtropical climate, with relatively heavy rainfall. It was crucial to capture the critical period of construction during the dry season. In the experimental study conducted in this article, factors such as low permeability coefficient, fast construction speed, and drainage boundary conditions of red clay were considered. Unconsolidated fast shear tests were adopted with a shear rate of 0.800 mm/min to obtain the unconsolidated and undrained strength index, which was used for roadbed stability analysis and bearing capacity verification. In actual engineering construction, with the passage of time and the influence of various external environmental factors, the pore pressure of the roadbed will gradually dissipate, and the roadbed soil will gradually consolidate. Most cases belong to “partial consolidation”. The use of unconsolidated and undrained strength indicators to guide engineering construction was biased toward safety.
(3) The size of the specimen is a factor that needs to be considered when conducting experimental research. The red clay sample used in this study was a cylindrical body with a diameter of 61.8 mm and a height of 20 mm. The small-sized sample will damage the structure of the soil to varying degrees during the preparation process, and it is more susceptible to disturbance, which affects the accuracy of the test results. Therefore, controlling the state of the experiment and effectively reducing the size effect brought by the sample during the direct shear test of remolded red clay was the key to obtaining correct results. Further research was needed to investigate the influence of specimens of different sizes on the test results. In addition, the shear strength of red clay was essentially the result of the combined effect of moisture content and pore structure. Research has shown that changes in moisture content can cause changes in the pore structure. However, this article only focuses on the independent study of compaction degree and moisture content, without considering the coupling effect of the two factors. At the same time, different sample preparation methods and stress states will have an impact on the test results. Therefore, further in-depth research is needed on the evolution of soil shear strength, moisture content, and compaction degree.
(4) As for the influence of compaction degree on the shear strength of red clay, the experimental results show that the cohesion value significantly decreases with increasing water content. The reason can be considered from the perspective of the interaction between soil and water: water in soil exists in the form of a bound water film around particles in the soil, which includes a strongly bound water film and a weakly bound water film. The water molecules in the strongly bound water film cannot move, whereas those in the weakly bound water film can move, providing lubrication for the relative movement between soil particles. As the moisture content increased, there was an increasing number of water molecules in the soil in the form of weakly bound water films and more free water. These water pressures tend to separate soil particles, and as the moisture content increases, the chances of particle interlocking decrease. Therefore, the strength generated by interlocking decreases, and the combined effect of the two leads to a significant decrease in cohesion with an increase in moisture content. In addition, this article only focuses on the study of red clay in the Guangxi region, and the relationship between soil strength, moisture content, and compaction may vary among different regions. According to the experimental results, when the moisture content ω was 15% and 18% at 50 kPa, the difference in shear strength was small. This may be due to the small vertical pressure, which causes the upper and lower boxes to move in a staggered manner during the shear process, resulting in continuous changes toward principal stress and uneven distribution of shear stress and strain.
(5) Regarding the influence of moisture content on the shear strength of red clay, as the moisture content increases, the internal friction angle slightly decreases. In addition, as the water content increases, the pores mainly increase in the form of capillary water, causing the water film effect of bound water to gradually weaken, resulting in a gradual decrease in the water film bonding force. At the same time, the bonding effect between particles mainly depends on the soil particle minerals. An increase in water content will cause the bonding material between particles to dissolve, and the bonding effect will gradually be lost, resulting in a decrease in cohesion. Therefore, it can be concluded that the higher the moisture content, the smaller the shear stress of compacted red clay [36]. Regarding the influence of compaction degree on the shear strength of red clay, an increase in compaction degree leads to an increase in shear stress, which can be explained from two aspects: cementation and pore ratio. First, as the compaction degree increases, the contact between soil particles becomes closer, leading to an enhanced interlocking effect and an increase in shear resistance and bonding effect. Second, the compaction degree increases and the pore ratio decreases. The water in the soil mainly exists in the form of adsorbed water film water, with relatively less capillary water. However, water film water is immovable, which is conducive to enhancing the interaction between water and soil particles. Based on the above analysis, it can be concluded that the increase in compaction enhances the shear stress [37].
(6) According to the comparison of the shear strength indices of soil samples under different working conditions, the influence of water content control on the *c* and *φ* is obviously greater than that of compaction degree control, and it is difficult to reach a compaction degree of 93% during soil preparation. Choosing compaction degree as the control index of the project in the actual process of the project will not only increase the construction difficulty of the project and affect the construction progress, but the required compaction machinery will further increase the construction cost and increase the project investment. Therefore, the soil moisture content should be used as the control index to guide the construction.

## 6. Conclusions

Relying on the project of the Nanning Bobai Nabu Section of the Nanning Zhanjiang Expressway in the Guangxi Zhuang Autonomous Region, this study uses high-liquid-limit red clay to prepare soil samples with different water contents (15%, 18%, 21%, 24%) and compaction (86%, 90%, 93%). The liquid plastic limit test, compaction test, ICP-OES, and XRD were used to explore its basic physical properties, and the direct shear test was used to explore the influence of compaction degree and water content on the shear strength of high-liquid-limit red clay. The results show that:

(1) Using the liquid plastic limit test, compaction test, ICP-OES, and XRD, it can be determined that the liquid limit of the soil sample at K21 + 100 is 57%, the plastic limit is 26.5%, the plasticity index is 30.5, the optimal water content is 15.3%, the maximum dry density is 1.82 g/cm^3^, and the contents of Al, Fe, and K are high.
(2) Under the same compaction degree, the shear stress of the soil sample shows an obvious increasing trend with an increase in normal stress, and the greater the water content, the smaller the shear stress. When the normal stress is 50 or 100 kPa and the compaction degree is not greater than 0.9, the difference in shear strength between 15% and 18% water content is very small. However, with an increase in normal stress or compaction degree, the shear strength of a sample with 15% water content will be higher than that of a sample with 18% water content. The greater the normal stress, the greater the water content, and the greater the weakening effect on the soil shear strength.
(3) At the same moisture content, the shear stress of the soil sample shows an increasing trend with an increase in compaction degree, and the greater the compaction degree, the greater the shear stress of the soil sample. For samples with water contents of 15% and 18%, the compaction degree has a significant impact on shear strength, but for samples with water contents of 21% and 24%, the change in compaction degree has a small impact on the change in shear strength. In the case of a certain water content, the cementation between soil particles will gradually increase with an increasing compaction degree, and the shear strength will show an upward trend.
(4) The *c* and *φ* of samples increase with an increase in the compaction degree, but an increase in *c* is affected by the water content. Under the same compaction degree, when the sample is near the optimal moisture content, the *c* is the largest, and the *φ* of the soil sample decreases with increasing moisture content. Under the same water content, the *φ* shows a certain increasing trend with an increasing compaction degree. When the soil is saturated, the free water content between soil particles increases significantly and the *c* decreases. When the soil moisture content is low, the soil particles can directly contact and closely occlude, and the shear strength is low.

## Figures and Tables

**Table 1:** Liquid plastic limit index of sample.

	Stake Number	K18 + 480	K21 + 100	K21 + 700	K22 + 980
Index					
Plastic limit		20.5	26.5	16.7	20.5
Liquid limit		44.6	57	32.9	45.4
Plasticity index		24.1	30.5	16.2	24.9
Soil texture		Low-liquid-limit clay	High-liquid-limit clay	Low-liquid-limit silt	Low-liquid-limit clay

**Table 2:** Test results of inductively coupled plasma optical emission spectrometer.

Element	Content (mg/L)	Element	Content (mg/L)
Ag	<0.0001	Mn	0.012
Al	8.69	Mo	<0.0001
As	0.0016	Na	0.033
Ba	0.018	Ni	0.0017
Be	0.0001	P	0.0066
Ca	0.019	Pb	0.0025
Cd	<0.0001	S	0.0056
Ce	0.0074	Sb	0.0003
Co	0.0008	Sc	0.0013
Cr	0.0050	Se	----
Cu	0.0017	Sn	<0.0001
Fe	3.56	Sr	0.0014
Hg	----	Ti	0.39
K	1.34	V	0.0099
La	0.0043	Y	0.0027
Li	0.0018	Zn	0.0043
Mg	0.60	Zr	0.013

**Table 3:** Compaction test results.

	Index	Moisture Content (%)	Dry Density (g/cm^3^)
Number			
1		9.5	1.75
2		11.4	1.78
3		12.8	1.80
4		15.2	1.83
5		19.2	1.75
6		24.4	1.62
7		26.0	1.55

**Table 4:** Control table of direct shear indices.

	Index	Compaction Degrees (%)	Water Content (%)	Normal Stress (Kpa)
Number				
1		86	15	50, 100, 150, 200
2		86	18	50, 100, 150, 200
3		86	21	50, 100, 150, 200
4		86	24	50, 100, 150, 200
5		90	15	50, 100, 150, 200
6		90	18	50, 100, 150, 200
7		90	21	50, 100, 150, 200
8		90	24	50, 100, 150, 200
9		93	15	50, 100, 150, 200
10		93	18	50, 100, 150, 200
11		93	21	50, 100, 150, 200
12		−93	24	50, 100, 150, 200

**Table 5:** Shear strength indices of red clay under different working conditions.

Water Content (%)	K = 86%		K = 90%		K = 93%	
	Cohesion *c* (kPa)	Internal Friction Angle *φ* (°)	Cohesion *c* (kPa)	Internal Friction Angle *φ* (°)	Cohesion *c* (kPa)	Internal Friction Angle *φ* (°)
15	82.6	32.2	120.2	32.5	170.2	32.9
18	92.6	23.9	122	24.6	136.45	28.2
21	64.9	21.2	75.55	24.8	84.6	26.3
24	25.95	21.5	28	22.8	35.2	24

## Data Availability

Data are contained within the article.

## Symbol Description

Symbol	Significance	Unit (Dimension)
*w*	Mass moisture content	%
*K*	Compaction degree	%
*m*	Quality of soil	g
*ρ_d_*	Actual dry density	g/cm^3^
*ρ_dmax_*	Maximum dry density	g/cm^3^
*ρ_w_*	Wet density	g/cm^3^
